# Deciphering the Roles of Metformin in Alzheimer’s Disease: A Snapshot

**DOI:** 10.3389/fphar.2021.728315

**Published:** 2022-01-27

**Authors:** Wang Liao, Jiaxin Xu, Bo Li, Yuting Ruan, Tian Li, Jun Liu

**Affiliations:** ^1^ Department of Neurology, The Second Affiliated Hospital of Guangzhou Medical University, Guangzhou, China; ^2^ Department of Neurology, Sun Yat-sen Memorial Hospital, Sun Yat-sen University, Guangzhou, China; ^3^ Department of Orthopedics, Sun Yat-sen Memorial Hospital, Sun Yat-sen University, Guangzhou, China; ^4^ Department of Rehabilitation Medicine, The Second Affiliated Hospital of Guangzhou Medical University, Guangzhou, China; ^5^ School of Basic Medicine, Fourth Military Medical University, Xi’an, China

**Keywords:** metformin, Alzheimer’s disease, insulin, AMPK, neuroinflammation

## Abstract

Alzheimer’s disease (AD) is a prevalent neurodegenerative disease predominantly affecting millions of elderly people. To date, no effective therapy has been identified to reverse the progression of AD. Metformin, as a first-line medication for Type 2 Diabetes Mellitus (T2DM), exerts multiple beneficial effects on various neurodegenerative disorders, including AD. Evidence from clinical studies has demonstrated that metformin use contributes to a lower risk of developing AD and better cognitive performance, which might be modified by interactors such as diabetic status and APOE-ε4 status. Previous mechanistic studies have gradually unveiled the effects of metformin on AD pathology and pathophysiology, including neuronal loss, neural dysfunction, amyloid-β (Aβ) depositions, tau phosphorylation, chronic neuroinflammation, insulin resistance, impaired glucose metabolism and mitochondrial dysfunction. Current evidence remains ambiguous and even conflicting. Herein, we review the current state of knowledge concerning the mechanisms of metformin in AD pathology while summarizing current evidence from clinical studies.

## Introduction

Alzheimer’s disease (AD) is a neurodegenerative disease characterized by a progressive decline of cognitive functions, such as memory and learning, sometimes accompanied by behavioral and psychological symptoms (Apostolova, 2016). With a high morbidity, disability and fatality rate, AD significantly increase global healthcare burden. Unfortunately, its neuropathological hallmarks, amyloid-β (Aβ) plaques and neurofibrillary tangles (NFTs) appear decades before the occurrence of cognitive impairment (Dubois et al., 2014). To date, no effective therapy has been identified to reverse or slow down the progression of AD and improve its clinical outcomes (Schneider et al., 2014). Therefore, early identification of people at higher risk of developing AD and early intervention are now the most practical strategies to reduce AD burden. A broader perspective on the treatment is also urgently needed.

Increasing evidence suggests a positive association between Type 2 Diabetes Mellitus (T2DM) and the risk of developing AD (Schilling, 2016; Yu et al., 2020). Impaired insulin signaling and glucose metabolism, two key mechanisms involved in T2DM, have been found to contribute to AD pathogenesis and progression (Gupta et al., 2011; Kandimalla et al., 2017; Benedict and Grillo, 2018; Boccardi et al., 2019). Researchers have even proposed the term “Type-3-Diabetes” for AD due to the significant association (Kandimalla et al., 2017), which implies the potential of anti-diabetic medicine to prevent or treat AD. Intranasal insulin has been considered as a promising candidate for AD prevention and treatment due to its safety and current encouraging clinical evidence on its effectiveness (Harris et al., 2020; Hallschmid, 2021). As for other anti-diabetic drugs, including dipeptidyl peptidase (DPP)-IV inhibitors and Glucagon-like peptide-1 (GLP-1) analogues, they were found to decrease neuroinflammation, Aβ load and tau phosphorylation in experimental studies, while clinical evidence is still lacking (McClean et al., 2011; Yang et al., 2013; Kosaraju et al., 2017; Boccardi et al., 2019).

Metformin, one of the first-line medications for T2DM, is a biguanide that lowers plasma glucose concentration mainly by improving insulin resistance and suppressing gluconeogenesis in the liver. Recent studies have further discovered its anti-inflammatory, anti-apoptotic and anti-oxidative effects (Li et al., 2020; Li et al., 2021; Ott et al., 2021; Tanaka et al., 2021). It is also confirmed that metformin can rapidly cross the blood-brain barrier (BBB) and accumulate in different brain regions (Łabuzek et al., 2010), influencing the central nervous system (CNS) (El-Mir et al., 2008). Metformin is now regarded as a magic drug that might benefit various diseases, including AD (Markowicz-Piasecka et al., 2017; Lv and Guo, 2020). Current studies on the effects of metformin on AD and the underlying mechanisms remain limited and controversial. It is also necessary to take its adverse effects into consideration before regarding metformin as a candidate for AD prevention and early-stage treatment. Moreover, it is important to confirm the cognitive safety of long-term metformin use in T2DM patients. The present review is to summarize the current knowledge on this topic and proposes promising directions for further research.

## Search Strategy and Selection Criteria

We initially searched PubMed from inception to June 2021 for English-only publications using predefined search terms for metformin and Alzheimer’s disease. We also reviewed the reference list of relevant articles. We included published observational studies and clinical trials exploring the cognitive effect of metformin on people with T2DM or cognitive impairment. As for preclinical studies, we included *in vitro* studies and *in vivo* studies using AD or diabetic or wild-type mouse models. When summarizing the evidence, further search was conducted to collect background information on the mentioned underlying mechanisms such as the association of AMP-activated protein kinase (AMPK) (Hsu et al., 2021) with metformin and AD.

## Mechanisms Linking Metformin With AD

### The Effects of Metformin on Neuronal Loss and Neural Dysfunction

Metformin treatment was shown to inhibit neuronal loss, the direct cause of cognitive deficits in AD, by promoting neurogenesis and inhibiting pathological neuronal apoptosis in the hippocampus of amyloid precursor protein/presenilin-1 (APP/PS1) mice (West et al., 1994; Ou et al., 2018). Similarly, Chen and others reported that metformin could ameliorate Aβ-induced apoptosis in hippocampal neurons by suppressing c-Jun N-terminal kinase (JNK) activation (Chen B. et al., 2016). Another *in vitro* study demonstrated that in db/db mice, metformin treatment could suppress the activation of caspase-3, one of the essential mediators of apoptosis (Chen F. et al., 2016). In human neural stem cells (hNSCs), co-treatment with metformin restored the Aβ-induced increase in caspase 3/9 activity and cytosolic cytochrome c released from mitochondria in an AMPK-dependent manner, probably leading to a suppression of apoptosis (Chiang et al., 2016). Metformin was also found to decrease apoptotic cell death induced by Aβ_25-35_ in SH-SY5Y cells (Li et al., 2019). However, Picone et al. observed promoted neuronal apoptosis by metformin in the cortex of C57B6/J mice (Picone et al., 2016). Further preclinical studies are warranted to probe the underlying mechanisms and the safety of metformin among nondiabetic and non-AD subjects.

Protein kinase C (PKC) has been known to affect insulin function and memory formation by promoting synaptogenesis (Nelson et al., 2008). Wang et al. showed that metformin enhanced the genesis of human and rodent neurons and promoted spatial memory formation by activating an atypical PKC-CREB-binding protein (aPKC-CBP) signaling pathway in neural precursors (Wang et al., 2012). Furthermore, Syal et al. reported that the activated aPKC-CBP pathway by metformin treatment could reverse impairment of neurogenesis and spatial memory in the 3xTg-AD mouse model by decreasing the expression of monoacylglycerol lipase (Mgll), a hydrolase-producing arachidonic acid (ARA) precursor pool, through degrading endocannabinoid 2-arachidonoyl glycerol (2-AG). Mgll was further considered as a potential biomarker to help identify metformin-responsive AD patients (Mulvihill and Nomura, 2013; Syal et al., 2020).

Metformin was also found to markedly increase the expression of neurotrophic and synapse-related factors (Bdnf, Ngf and Syp) in the brain of APP/PS1 mice (Lu et al., 2020). Pilipenko reported that metformin could restore hippocampal synaptic plasticity in streptozocin (STZ)-induced sporadic AD rats (Pilipenko et al., 2020). In APP/PS1 mice, metformin was shown to improve synaptic defects, including spine loss, inhibit basal synaptic transmission and decrease the expression of surface GluA1 by suppressing the overactivation of cyclin-dependent kinase 5 (Cdk5), which play a critical role in synaptic plasticity (Sheng et al., 2016; Wang et al., 2020). Chen et al. found that metformin potentiated glutamatergic synaptic transmission onto the hippocampal CA1 pyramidal neurons in C57BJ/6 mice (Chen et al., 2020). This promotion by metformin may improve the dysfunction of glutamatergic synapses, which appear in the early stage of AD (Benarroch, 2018).

### The Effects of Metformin on Aβ Pathology

**FIGURE 1:**
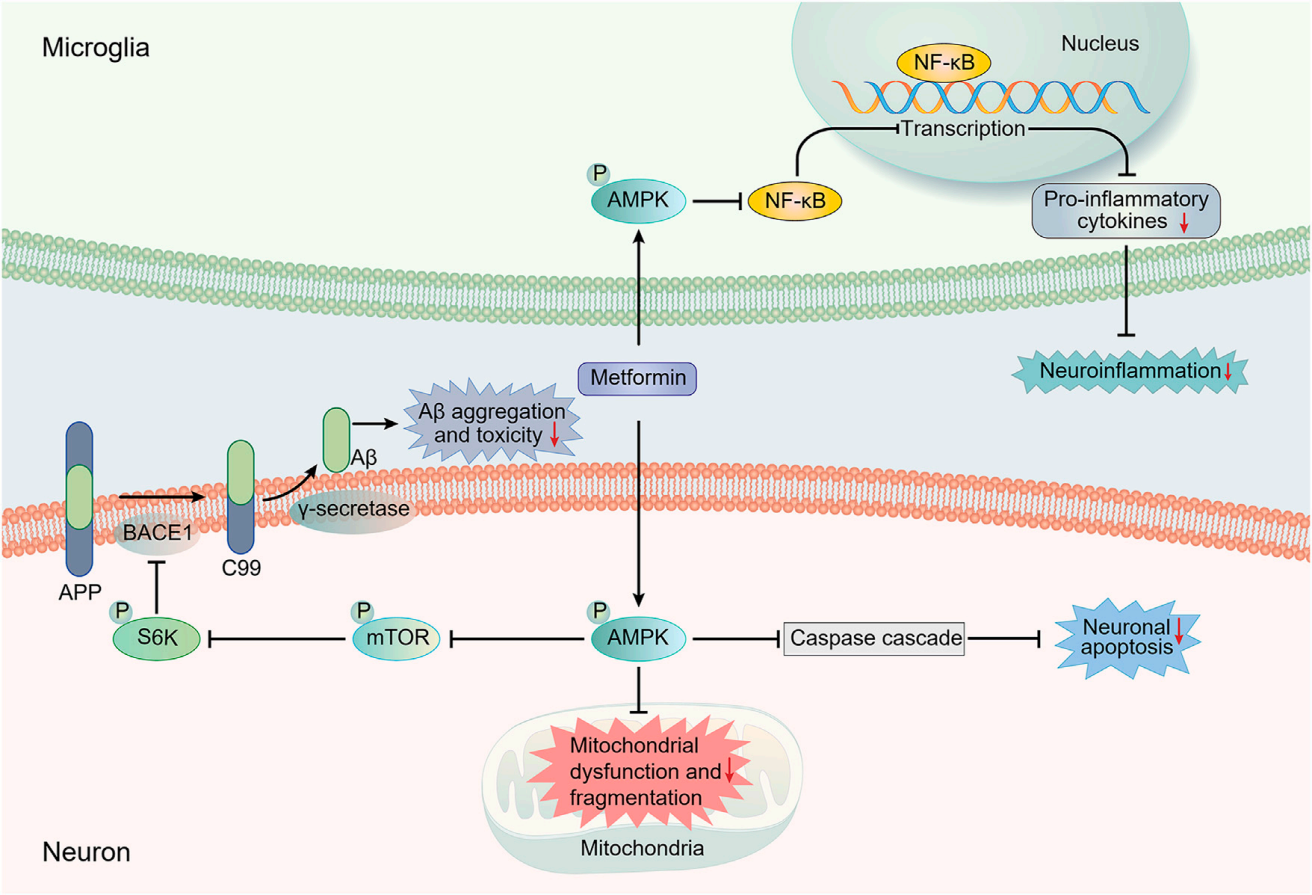
Main potential AMPK-dependent molecule mechanisms involved in the neuroprotective effects of metformin against AD pathology. Most presented mechanistic studies have implied an AMPK-dependent neuroprotective action of metformin against AD. The regulation of AMPK/mTOR/S6K/BACE1 signaling pathway by metformin can result in a reduction in Aβ production, thus decreasing Aβ toxicity. AMPK activation can also suppress the activation of the NF-κB pathway, which is regarded as the central signaling pathway involved in AD neuroinflammation. Moreover, the increased activation of caspase cascade, which is involved in the apoptosis-mediated neurodegeneration, and the impairment of mitochondrial function and morphology induced by Aβ can be inhibited by metformin in an AMPK-dependent manner. AMPK, AMP-activated protein kinase; APP, amyloid precursor protein; Aβ, amyloid-β; BACE1, β-site APP cleaving enzyme-1; mTOR, mechanistic target of rapamycin; NF-κB, Nuclear factor-kappa B; S6K, ribosomal S6 kinase.

The insulin-degrading enzyme (IDE), the protease that cleaves β-forming peptides such as insulin and Aβ, plays a major role in the crosstalk between T2DM and AD (Kurochkin et al., 2018). Lu et al. proposed that the reduction of Aβ deposition in APP/PS1 mice after metformin usage might be mediated by the increased level of IDE. They also observed a slightly decreased expression of β-secretase BACE1 (one of the key enzymes responsible for the cleavage of APP to produce Aβ) and no significant change in the expression of other Aβ-related secretases (A Disintegrin and metalloproteinase domain-containing protein 10 (ADAM10) and PS1) and transporters (lipoprotein receptor-related protein (LRP1) and receptor for advanced glycation end products (RAGE)) (Lu et al., 2020; Hampel et al., 2021). Ou et al. reported a reduction in BACE1 expression and amyloid plaque accumulation in the hippocampus and cortex of APP/PS1 mice treated with metformin. The reduction was mediated through regulating the AMPK/mTOR/S6K/BACE1 signaling pathway (Ou et al., 2018). It is noteworthy that AMP-activated protein kinase (AMPK) is critical in regulating the effect of metformin on AD pathology (Figure 1) (Ou et al., 2018). Previous studies have demonstrated the ability of metformin to activate AMPK thus exerting various effects, including a possible neuroprotective effect on AD pathology (Gupta et al., 2011; Foretz et al., 2014; Yang et al., 2020). As for Aβ transport, Chen et al. reported that metformin could decrease RAGE expression and Aβ influx across the BBB, restoring the abnormal Aβ transport in db/db mice (Chen F. et al., 2016).

However, a previous *in vitro* study showed that metformin alone exerted no significant effect on IDE but up-regulated AMPK-dependent BACE1 expression to increase Aβ generation in N2a695 cells overexpressing APP. A combination with insulin reversed the deleterious effect of metformin, suggesting an important regulatory role of insulin in metformin function (Chen et al., 2009; El Massry et al., 2020). Similarly, overexpression of APP and PS1 in LAN5 neuroblastoma cells treated with metformin was suppressed by insulin. In C57B6/J mice, metformin activated AMPK and increase the levels of APP and BACE1, thus promoting the production of Aβ in the cortex (Picone et al., 2015). Metformin was also found to directly interact with Aβ, leading to the alteration of its aggregation profiles, including a reduction of harmless mature fibrils and stabilization of toxic prefibrillar oligomers, suggesting an increase in Aβ toxicity (Picone et al., 2016).

### The Effects of Metformin on Tau Pathology

The underlying mechanisms behind the complex effects of metformin on tau pathology remain elusive. Metformin played paradoxical roles in tauopathy in the brain of mutant human tau (P301S) transgenic mice, including phosphorylation-suppressing and pro-aggregation effects; the former benefit seemed to be weakened by the latter harm (Barini et al., 2016). Mostafa et al. observed that metformin decreased the level of p-tau in the hippocampus and cortex of mice with scopolamine-induced cognitive impairment, probably through increasing the level of phosphorylated Akt while decreasing that of total Akt (Mostafa et al., 2016). Kickstein et al. also verified the ability of metformin to decrease tau phosphorylation at Ser202 both *in vitro* and *in vivo*, which was dependent on the activation of the mTOR/protein phosphatase 2A (PP2A) signaling pathway and unrelated to AMPK activation (Kickstein et al., 2010). Metformin injection for 18 weeks attenuated the increase of phospho-tau phosphorylated at Ser396 and Thr231 in the hippocampus of obese, leptin-resistant mice with AD-associated brain changes through suppressing the activation of JNK instead of attenuating the decrease of PP2A (Li et al., 2012). Metformin was also found to restrict the spreading of tau pathology and reduce Aβ plaques in APP/PS1 mice injected with tau seeds, probably through promoting microglial autophagy of the two pathologies (Chen et al., 2021). However, Zhang et al. reported that chronic metformin administration increased tau phosphorylation in old ApoE-target replacement (TR) mice, probably through increasing the phosphorylation of GSK-3β at Tyr216 (Zhang et al., 2019). Similarly, Kuhla et al. also reported a metformin-induced increase in tau phosphorylation in ApoE deficient (ApoE^−/−^) mice, a mouse model of tauopathy (Kuhla et al., 2019). Further research is needed to confirm whether ApoE genotypes can modify the effects of metformin.

### The Effects of Metformin on Neuroinflammation

Accumulating data have revealed the ability of metformin to modulate chronic neuroinflammation (one of the core pathologies in AD). This includes sustained activation of immune cells such as microglia and increased proinflammatory cytokines (Kinney et al., 2018). Previous studies have proposed that metformin could effectively reduce the levels of proinflammatory cytokines such as tumor necrosis factor-alpha (TNF-α), interleukin 1 beta (IL-1β) and IL-6, in the brain of APP/PS1 mice, which could be attributed to the activation of AMPK/P65 NF-κB signaling pathways observed in the hippocampus (Ou et al., 2018; Lu et al., 2020). Nuclear factor-kappa B (NF-κB) is a well-known pro-inflammatory transcription factor involved in abnormal neuroinflammation in AD (Thawkar and Kaur, 2019). Ou et al. also observed decreased accumulation of activated microglia and astrocytes around Aβ plaques in metformin-treated APP/PS1 mice (Ou et al., 2018). In a mouse model with STZ-induced sporadic AD, microgliosis and astrogliosis were also improved by metformin (Pilipenko et al., 2020). Interestingly, Chen et al. reported enhanced microgliosis around Aβ plaques, promoted microglial phagocytosis and ameliorated autophagy of tau aggregates in metformin-treated APP/PS1 mice initially injected with tau aggregates (Chen et al., 2021). It has been suggested that moderate activation of autophagy is critical in the clearance of protein aggregates, thus delaying progression in the early stage of AD (Li et al., 2017). The difference in microglia activation might be associated with injected tau aggregates stimulation.

### The Effects of Metformin on Insulin Resistance and Glucose Metabolism

Mounting evidence has supported a robust correlation between insulin resistance and neurodegenerative changes in AD, such as Aβ accumulation and tau phosphorylation (Kellar and Craft, 2020). It is well established that metformin can alleviate hyperinsulinemia and insulin resistance, secondarily, improving metabolic dysfunction (Kitabchi et al., 2005). Considering the ability of metformin to cross the BBB, it is reasonable to speculate that metformin may directly improve AD-related impairment of insulin signaling and glucose metabolism in the CNS. Gupta et al. reported that metformin treatment could alleviate the insulin resistance and AD-related pathological changes induced by hyper-insulinemic exposure in neuronal cell lines (Gupta et al., 2011).

Glucose hypometabolism has been acknowledged as a key pathophysiological characteristic of AD (Daulatzai, 2017). Metformin effectively increased brain glucose uptake in APP/PS1 mice (Lu et al., 2020). Glucose transporters (GLUTs) deficiency is one of the main causes of the decreased glucose metabolism. GLUT-1 is mainly expressed in BBB endothelial cells and thus plays a crucial role in the glucose transport across the BBB. GLUT-3 is expressed in neurons, and thus it is in charge of glucose uptake into neurons (Shah et al., 2012). Glycogen synthase kinase-3 (GSK-3) is associated with the impairment of glucose tolerance (Pearce et al., 2004). In rats with STZ-induced sporadic AD, metformin elevated the density of GLUT-1 and GLUT-3 but decreased the density of GSK-3 in the hippocampus and cortex, suggesting a normalization of brain glucose transport and uptake (Pilipenko et al., 2020). Niccoli et al. reported that metformin could mimic the impact of overexpressed GLUT-1 to increase neuronal glucose uptake in the adult-onset *Drosophila* model (Niccoli et al., 2016). Thus, metformin treatment could considerably restore the impaired glucose metabolism in the brain.

### Other Effects of Metformin on AD

The anti-oxidative property of metformin also plays a vital role in ameliorating AD pathology. In APP/PS1 mice, the increase in superoxide dismutase (SOD) activity and malondialdehyde (MDA) level was attenuated by metformin administration (Lu et al., 2020). In rats with scopolamine-induced cognitive impairment, metformin decreased the level of nitric oxide (NO) and MDA in the frontal cortical and hippocampal tissues (Mostafa et al., 2016). Metformin was also found to inhibit the elevated levels of inflammation and endoplasmic reticulum (ER) stress by high glucose treatment through suppressing the interaction between AMPKα and caveolin1 in primary rat astrocytes (Wang et al., 2021).

Mitochondrial dysfunction has been identified as an early event of AD (Desler et al., 2018). Exposure to Aβ could result in an impairment of mitochondrial function and morphology in hNSCs. Co-treatment with metformin, however, was found to protect against the impairment, which was dependent on AMPK activation (Chiang et al., 2016). An *in vivo* study using SAMP8 mice of sporadic AD reported that metformin decreased the level of APPc99 and pTau404 expression, which was associated with improved mitochondrial function in previous studies (Reddy, 2011; Pera et al., 2017; Farr et al., 2019). However, metformin treatment for 3 months was found to increase the levels of the translocase of the outer membrane 40 (TOM40), hexokinase I (HKI) and voltage-dependent anion-selective channels 1 (VDAC) and influence their conformation in the cortex region of C57B6/J mice, suggesting impairment of mitochondrial permeability transition pores and membrane channels, thereby leading to mitochondrial dysfunction (Picone et al., 2016).

Growing evidence has indicated an anti-degenerative benefit from the inhibition of acetylcholinesterase (AChE), the enzyme secreted into the synaptic cleft to inactivate acetylcholine (ACh) by cholinergic neurons (Krishnan et al., 2003; Ferreira-Vieira et al., 2016; Cavedo et al., 2017; Moss, 2020). Gupta found that metformin could inhibit the increase of AChE activity by 34.9% in insulin-resistant neuroblastoma cells (Gupta et al., 2011). Bhutada et al. reported a decrease in the activity of choline esterase (ChE) after chronic metformin treatment in the hippocampus of rats with STZ-induced diabetes (Bhutada et al., 2011). In the rat model of STZ-induced sporadic AD, metformin inhibited AChE activity in the hippocampal CA1 and CA3. Interestingly, metformin alone increased AChE activity in similar regions of non-AD rats (Pilipenko et al., 2020), probably implying an interaction between metformin and some AD-related pathophysiological change induced by STZ.

## Clinical Evidence

Current epidemiological studies regarding the effects of metformin on the risk of AD remain still scanty and contradictory (Table 1). Most studies set the outcome as the onset of all-cause cognitive dysfunctions such as dementia and the participants as T2DM patients. A meta-analysis conducted in 2018 have shown that treatment with metformin could effectively lower the risk of subsequent dementia by 34% in elderly people with T2DM (Campbell et al., 2018). Consistently, a recent longitudinal study among community-dwelling diabetic patients in Finland suggested that long-term high-dose metformin use could decrease the risk of AD in elderly diabetic patients (Sluggett et al., 2020). Similarly, Wu et al. observed an association between metformin treatment and less memory decline over time in cognitively normal T2DM patients. Interestingly, this association was not observed among the APOE ε4 carriers, which implied an interaction between APOE ε4 carrier status and metformin (Wu et al., 2020). Orkaby et al. found that the protective association between metformin and dementia risk could also be modified by age, race, level of HbA1c and renal function. Compared with sulfonylurea, metformin could significantly lower the risk of dementia only among T2DM patients aged 75 or younger who were white, or had good renal function or HbA1C values ≥ 7% (Orkaby et al., 2017). These findings have implied that it is necessary to identify the subgroups of people that can benefit from metformin use for the prevention of cognitive impairment.

**TABLE 1:** Representative clinical studies on the effects of metformin on AD. AD, Alzheimer’s disease; MCI, mild cognitive impairment; T2DM, type 2 diabetes mellitus.

Authors	Study design	Data source	Participants	Conclusions
Sluggett et al., 2020	Nested case-control study	The National Medication Use and Alzheimer’s disease (MEDALZ) study	Community-dwelling Finns with diabetes diagnosed ≥3 years before AD (n = 9,862); Diabetic Controls matched by age, sex, and diabetes duration (n = 19550)	Metformin use (ever use) was not associated with incident AD Long-term and high-dose metformin use was associated with a lower risk of incident AD
Hsu et al., 2011	Cohort study	The National Health Insurance database in Taiwan	Nondemented T2DM patients aged 50 or older taking metformin only (n = 1864)	Compared with no medication, metformin alone reduced the risk of developing dementia by 24%, combined therapy with sulfonylureas by 35%
			taking sulfonylureas only (n = 3,753)	
			taking both (n = 9,257)	
			taking no anti-diabetic drug (n = 10519)	
Orkaby et al., 2017	Cohort study	The National Veterans Administration (VA) clinical and administrative database	US veterans aged 65 or older with T2DM but free of dementia new users of metformin (n = 17200)	In veterans <75 years of age, risk of incident dementia was lower among metformin users than sulfonylurea users
			new users of sulfonylurea (n = 11440)	
Cheng et al., 2014	Cohort study	The National Health Insurance database in Taiwan	Nondemented, nondiabetic participants aged 65 or older developing new-onset T2DM taking metformin alone (n = 1,033)	The risk effect of T2DM could be weakened by the use of metformin and sulfonylureas (no significant difference was shown between these two drugs)
			taking sulfonylureas alone (n = 796)	
			taking thiazolidinediones alone (n = 28)	
			not developing new-onset T2DM (n = 62311)	
			during the follow-up from January 2004 to December 2009	
Imfeld et al., 2012	Case-control study	The United Kingdom-based General Practice Research database	Individuals aged 65 or older with an incident diagnosis of AD (n = 7,086)	Long-term use of metformin was associated with a slight increase in the risk of developing AD, but no dose-effect relationship was observed
			without dementia and matched by age, sex, general practice, calendar time, and years of history (n = 7,086)	
Luchsinger et al., 2016	Randomized placebo-controlled clinical trial	N/A	Individuals aged 55–90 years with aMCI, a BMI of 25 kg/m^2^ or higher, without treated diabetes, randomized to take metformin 1,000 mg twice a day for 12 months (n = 40)	Metformin usage resulted in greater improvement than placebo in the total recall in the Selective Reminding Test as well as a statistically nonsignificant increase in plasma Aβ_42_
			matching placebo (n = 40)	
Koenig et al., 2017	Randomized placebo-controlled crossover study	N/A	Nondiabetic subjects with MCI or mild dementia due to AD, randomized to take metformin then placebo for 8 weeks each (n = 10)	Metformin treatment was significantly associated with improved executive functioning, and trends suggested improved learning/memory and attention by metformin
			placebo then metformin for 8 weeks each (n = 10)	
Wu et al., 2020	Observational study	The National Alzheimer’s Coordinating Center database	T2DM patients using a hypoglycemic medication with normal cognition (n = 1,192)	Metformin treatment exerted a memory benefit over time among cognitively normal people. There was no association between metformin and memory decline in AD, but metformin was associated with a greater decline in delayed memory specifically among APOE ε4 carriers
			taking metformin (n = 824)	
			with AD (n = 807)	
			taking metformin (n = 473)	
Moore et al., 2013	Observational study	The Primary Research in Memory (PRIME) clinics study, the Australian Imaging, Biomarkers and Lifestyle (AIBL) study of aging, and the Barwon region of southeastern Australia	Patients with AD (n = 480) patients with MCI (n = 187)	Metformin use was associated with impaired cognitive performance, which could be alleviated by vitamin B_12_ and calcium supplements
			cognitively intact individuals (n = 687)	

Metformin was also found to have a greater preventive effect than thiazolidinediones (Cheng et al., 2014). A combination of metformin and sulfonylurea could reduce the risk of dementia by 35% compared with no medication use, exhibiting better safety and efficacy (Hsu et al., 2011). A previous case-control study by Imfeld et al. reported an association between long-term metformin use and a slightly higher risk of developing AD, but no dose-effect relationship was observed (Imfeld et al., 2012). The cases and controls were not matched on diabetes diagnosis, which was in fact an important confounder in this study, likely leading to this inconsistent finding.

Several studies have also investigated whether metformin could exert a cognitive benefit in patients with cognitive impairment (Table 1). There have been two small-scale randomized clinical trials showing positive results. Luchsinger et al. found that metformin 1,000 mg twice a day for 12 months could result in greater improvement than placebo use in selective reminding test (SRT) as well as a statistically nonsignificant increase of plasma Aβ_42_ in overweight patients with amnestic mild cognitive impairment (aMCI) (Luchsinger et al., 2016). Another randomized crossover study patients with MCI or mild dementia due to AD suggested that metformin use could contribute to an improvement of executive function (Koenig et al., 2017). However, in the longitudinal study by Wu et al., no relationship was found between metformin treatment and longitudinal memory change among all T2DM patients with aMCI or AD dementia. Further analysis showed an association between metformin use and faster decline in delayed memory among the APOE ε4 carriers, implying that the adverse interaction between metformin and APOE-ε4 genotype or AD pathology might neutralize the neuroprotective effect of metformin itself (Wu et al., 2020). A small-scale cross-sectional study reported an association between metformin use and worse cognitive performance among diabetic patients, which might be mediated by vitamin B_12_ deficiency (Moore et al., 2013). The contradictions in the findings above might stem from the insufficient sample sizes of clinical trials and different diabetic status of participants.

In conclusion, current evidence has mainly suggested a cognitive benefit of metformin among cognitive normal T2DM patients while its cognitive effect on T2DM patients with AD seems negative. The underlying mechanisms of the adverse interaction between metformin and APOE-ε4 genotype or AD pathology need to be uncovered. Further works are warranted to identify the characteristics of people who benefit from metformin for the prevention or treatment of cognitive impairment. Large-scale, placebo-controlled randomized clinical trials are also needed to determine whether these effects can be generalized among population without T2DM. It will provide valuable insights into the underlying mechanisms if measuring AD biomarkers such as Aβ, phosphorylated tau (p-tau) and total tau (t-tau) in cerebrospinal fluid (CSF) and brain atrophy in future clinical research.

## Conclusions and Future Perspectives

This review mainly focuses on the clinical and mechanistic evidence on the effects of metformin on AD. The specific mechanisms linking T2DM to AD and the general pharmacological mechanisms are reviewed briefly.

Most clinical evidence has suggested that metformin, as a first-line glucose-lowering medication, helps lower the risk of AD among people with T2DM. Potential explanations include an improvement in the various macrovascular disorders associated with T2DM and the specific neuroprotective function of metformin reviewed above (Hsu et al., 2011; Cheng et al., 2014; Orkaby et al., 2017; Campbell et al., 2018; Sluggett et al., 2020; Wu et al., 2020). It is important to note that the effect of metformin on AD pathology is independent of glycemic control. However, current evidence failed to support the therapeutic role of metformin. Although most animal studies have suggested a neuroprotective role of metformin among APP/PS1 mice, clinical studies found no significant cognitive benefit of metformin among subjects with cognitive impairment due to AD (Moore et al., 2013; Wu et al., 2020).

Both preclinical and clinical studies have implied an interaction between metformin and ApoE carrier status (Kuhla et al., 2019; Zhang et al., 2019; Wu et al., 2020). Moreover, there are no studies probing the impact of metformin on non-diabetes subjects with normal cognitive function. It is necessary to identify the characteristics of people who get protection from metformin against AD. The modifiers such as APOE-ε4 genotype should also be taken into consideration when prescribing antidiabetic medication. It would be interesting to compare the effects generated by metformin and insulin treatment since there has been encouraging evidence suggesting the effectiveness of intranasal insulin treatment in AD (Boccardi et al., 2019). It is still unclear about the interactions between metformin and other common anti-AD drugs. Further investigation is needed to demonstrate whether their combination can be more effective in the fight against AD.

The poor oral absorption and gastrointestinal adverse effects significantly limited the clinical application of metformin. Researchers have attempted to design and synthesize prodrugs of metformin with higher lipophilicity to improve permeability and passive transcellular absorption. There have been several lipophilic sulfenamide prodrugs designed and shown to achieve better absorption (Huttunen et al., 2009; Huttunen et al., 2013). Furthermore, Rautio et al. synthesized novel sulfonamide prodrugs that would only be bioactivated by glutathione-S-transferase after oral absorption, which further improved the bioavailability of metformin (Rautio et al., 2014). Further studies are warranted to design new prodrug structures or other techniques making metformin reach the CNS more precisely.

After oral administration, metformin is delivered to the CNS *via* cerebral blood flow (CBF) and transported across the BBB. It is important to note that CBF reduction and the impairment of BBB integrity are early events occurred in AD pathogenesis, which may influence the uptake of metformin across the BBB (Kisler et al., 2017). Interestingly, a clinical trial by Koenig et al. showed that metformin might also affect CBF, as superior and middle orbitofrontal CBF was increased after 8 weeks of metformin treatment (Koenig et al., 2017). In Wistar rats, metformin was reported to efficiently cross the BBB and differently accumulate in brain regions, including hippocampus and frontal cortex after chronic oral administration for 3 weeks (Łabuzek et al., 2010). The patterns of metformin accumulation in different brain structures under AD pathology is still unclear. Further research is needed to confirm the appropriate dose and duration of metformin use required to produce sufficient protective effects against AD while causing less adverse effects.
